# Both Pseudomonas aeruginosa and Candida albicans Accumulate Greater Biomass in Dual-Species Biofilms under Flow

**DOI:** 10.1128/mSphere.00416-21

**Published:** 2021-06-23

**Authors:** Swetha Kasetty, Dallas L. Mould, Deborah A. Hogan, Carey D. Nadell

**Affiliations:** a Department of Biological Sciences, Dartmouth, Hanover, New Hampshire, USA; b Department of Microbiology and Immunology, Geisel School of Medicine at Dartmouth, Hanover, New Hampshire, USA; University of Georgia

**Keywords:** *Candida albicans*, *Pseudomonas aeruginosa*, artificial sputum, biofilm, confocal microscopy, cystic fibrosis, flow, image analysis, microfluidics, population dynamics, spatial ecology

## Abstract

Microbe-microbe interactions can strongly influence growth and biofilm formation kinetics. For Pseudomonas aeruginosa and Candida albicans, which are found together in diverse clinical sites, including urinary and intravenous catheters and the lungs of individuals with cystic fibrosis (CF), we compared the kinetics of biofilm formation by each species in dual-species and single-species biofilms. We engineered fluorescent protein constructs for P. aeruginosa (producing *mKO-κ*) and C. albicans (producing *mKate2*) that did not alter growth and enabled single-cell resolution imaging by live-sample microscopy. Using these strains in an optically clear derivative of synthetic CF sputum medium, we found that both P. aeruginosa and C. albicans displayed increased biovolume accumulation—by three- and sixfold, respectively—in dual-species biofilms relative to single-species biofilms. This result was specific to the biofilm environment, as enhanced growth was not observed in planktonic cocultures. Stimulation of C. albicans biofilm formation occurred regardless of whether P. aeruginosa was added at the time of fungal inoculation or 24 h after the initiation of biofilm development. P. aeruginosa biofilm increases in cocultures did not require the Pel extracellular polysaccharide, phenazines, and siderophores known to influence C. albicans. P. aeruginosa mutants lacking Anr, LasR, and BapA were not significantly stimulated by C. albicans, but they still promoted a significant enhancement of biofilm development of the fungus, suggesting a fungal response to the presence of bacteria. Last, we showed that a set of P. aeruginosa clinical isolates also prompted an increase of biovolume by C. albicans in coculture.

**IMPORTANCE** There is an abundance of work on both P. aeruginosa and C. albicans in isolation, and quite some work as well on the way these two microbes interact. These studies do not, however, consider biofilm environments under flow, and our results here show that the expected outcome of interaction between these two pathogens can actually be reversed under flow, from pure antagonism to an increase in biomass on the part of both. Our work also highlights the importance of cellular-scale spatial structure in biofilms for understanding multispecies population dynamics.

## INTRODUCTION

Microbial biofilm growth, even in monospecies contexts, involves the interplay of many biological and physical factors that are dynamic in space and time (1–3). In many natural environments, including numerous chronic infections, biofilms are multispecies mixtures whose collective properties and dynamics may be difficult to predict from those of each constituent’s monospecies biofilm growth. In the context of infection, the extent and kind of interactions among different biofilm-dwelling microbes also govern clinically relevant factors, such as drug resistance and virulence (4). For example, multispecies biofilm growth has been implicated in conjunctivitis (5), tooth decay (6), prosthesis and wound infections (7, 8), and respiratory diseases (9, 10). Clinical microbiologists are just starting to consider the multispecies nature of pathogenic biofilms and its implications for prevention and treatment (11).

Exemplars of chronic, multispecies biofilm infections are those that occur consistently in the lungs of patients with cystic fibrosis (CF), a genetic disorder in humans as a result of mutations in the cystic fibrosis transmembrane conductance regulator. Disruption of this protein’s function results in pathologies throughout the body including the accumulation of highly viscous mucus in the lungs, which hinders normal mucociliary clearance. As a result, bacterial and fungal pathogens that would otherwise be easily removed from healthy lungs instead accumulate and lead to chronic infections (12). Chronic CF lung infections are caused by diverse and metabolically flexible populations and consortia, and they are extremely recalcitrant to antibiotic and phagocytic clearance (13). While the ecology of the infecting species shapes the community and potentially has a profound influence on disease severity in the CF lung, it remains poorly understood (9). Given that the spatial interactions of pathogens can strongly affect disease outcome (14), we aimed to create an experimental model *in vitro* to investigate the biofilm formation kinetics of one or more species in coculture. Studies of multispecies biofilm formation and biofilm dynamics in general benefit tremendously from high-resolution imaging, which allows for studying the cell-length-scale behaviors and higher-order structures that contribute to the community’s cumulative growth, organization, and function. However, imaging live biofilms *in situ* is often difficult, if not impossible, in many natural contexts. A helpful strategy to mitigate this problem is to reconstitute key features of the *in situ* environment using an *in vitro* system that is more amenable to imaging.

Here, we chose to study Pseudomonas aeruginosa and Candida albicans as representatives of potentially interacting species in a polymicrobial CF infection, as both these species are commonly isolated from CF lung infections and believed to be important copathogens in patients (15). They are also thought to cooccur in other infection environments, including trauma wounds and surrounding urinary catheters (16). C. albicans is a polymorphic and opportunistic pathogen with the ability to form invasive hyphal filaments and drug-resistant biofilms (17). P. aeruginosa is another opportunistic pathogen with diverse virulence mechanisms, to which biofilm formation contributes directly and indirectly (18). P. aeruginosa*-*C. albicans interactions are well studied in liquid and agar colony models. Among the primary findings from this literature, P. aeruginosa has been shown to preferentially attach to and form biofilms on C. albicans hyphae in static culture, eventually killing them (19), but P. aeruginosa also inhibits the yeast-to-hyphal switch of C. albicans in liquid and agar colony cultures, enhancing C. albicans survival (20). Prior work has intimated a feedback loop whereby C. albicans produces ethanol, which increases biofilm formation, inhibits swarming motility, and enhances the production of antifungal phenazines on the part of P. aeruginosa (21, 22). These phenotypes in turn cause downregulation of the central pathway that induces hyphal growth and inhibit mitochondrial activity, stimulating further ethanol production by C. albicans (23). On the other hand, some *in vivo* experiments using a zebrafish model have indicated mutually enhanced virulence of the two species, suggesting that environmental shifts may have strong impacts on the properties of cocultures of these microbes (24). As local concentrations of metabolic products involved in interspecies interactions are determined by the relative and absolute abundances, it is critical to understand the dynamics of biofilm formation for each species in mixed culture.

Using engineered strains with novel fluorescent protein constructs and microfluidic culture with a modified synthetic sputum medium allowing for high-resolution imaging of C. albicans and P. aeruginosa, we show that their biofilm architecture, rates of biovolume accumulation, and total biovolume is higher for each species in coculture versus monococulture. Growth stimulation for either species was not observed in planktonic coculture conditions. This result is robust to different clinical strains of P. aeruginosa and a variety of deletion mutants lacking factors known to participate in P. aeruginosa*-*C. albicans interactions.

## RESULTS

### Biofilm profiles in mono- and dual-species culture.

We aimed to characterize the architecture of monospecies and dual-species biofilms of P. aeruginosa and C. albicans under flow in a medium that represents the chemical composition of CF sputum. Synthetic cystic fibrosis medium (SCFM2), developed and refined by the Whiteley group (25, 26), is a field standard for this purpose, but this medium is not optically clear due to the presence of reconstituted mucins. To generate an optically clear medium for imaging—and supported by data showing that P. aeruginosa does not degrade mucins itself (27)—we made a modified version of SCFM in which the major mucin glycans were substituted for mucin; we term this modified medium artificial sputum medium for imaging, or ASMi (see Materials and Methods). Each species’ growth profile was the same SCFM2 as it was in ASMi (see Fig. S1 in the supplemental material).

10.1128/mSphere.00416-21.1 FIG S1 Growth curves of (A) C. albicans and (B) P. aeruginosa in standard SCFM2 medium with reconstituted mucin and in ASMi medium containing only mucin sugars without full-length mucin polymers (*n* = 6). All error bars indicated are standard errors. Download FIG S1, TIF file, 2.6 MB. Copyright © 2021 Kasetty et al. 2021 Kasetty et al. https://creativecommons.org/licenses/by/4.0/ This content is distributed under the terms of the Creative Commons Attribution 4.0 International license.

P. aeruginosa and C. albicans were modified by allelic exchange to contain a chromosomal construct for constitutive expression of tandem, codon-optimized copies of *mKO-κ* (P. aeruginosa) or a single copy of *mKate2* (C. albicans) (see Materials and Methods). *mKO-κ* or *mKate2* was selected for these studies for their brightness and because they could be easily distinguished by fluorescence microscopy. The fluorescent protein expression constructs did not alter the growth rate of either species (Fig. S2).

10.1128/mSphere.00416-21.2 FIG S2 Growth curves in ASMi medium of (A) wild-type C. albicans CAI4 or C. albicans_ *mKate2* and (B) wild type P. aeruginosa PA14 or P. aeruginosa_ *mKO-κ* (*n* = 6). All error bars indicated are standard errors. Download FIG S2, TIF file, 0.4 MB. Copyright © 2021 Kasetty et al. 2021 Kasetty et al. https://creativecommons.org/licenses/by/4.0/ This content is distributed under the terms of the Creative Commons Attribution 4.0 International license.

**FIG 1:**
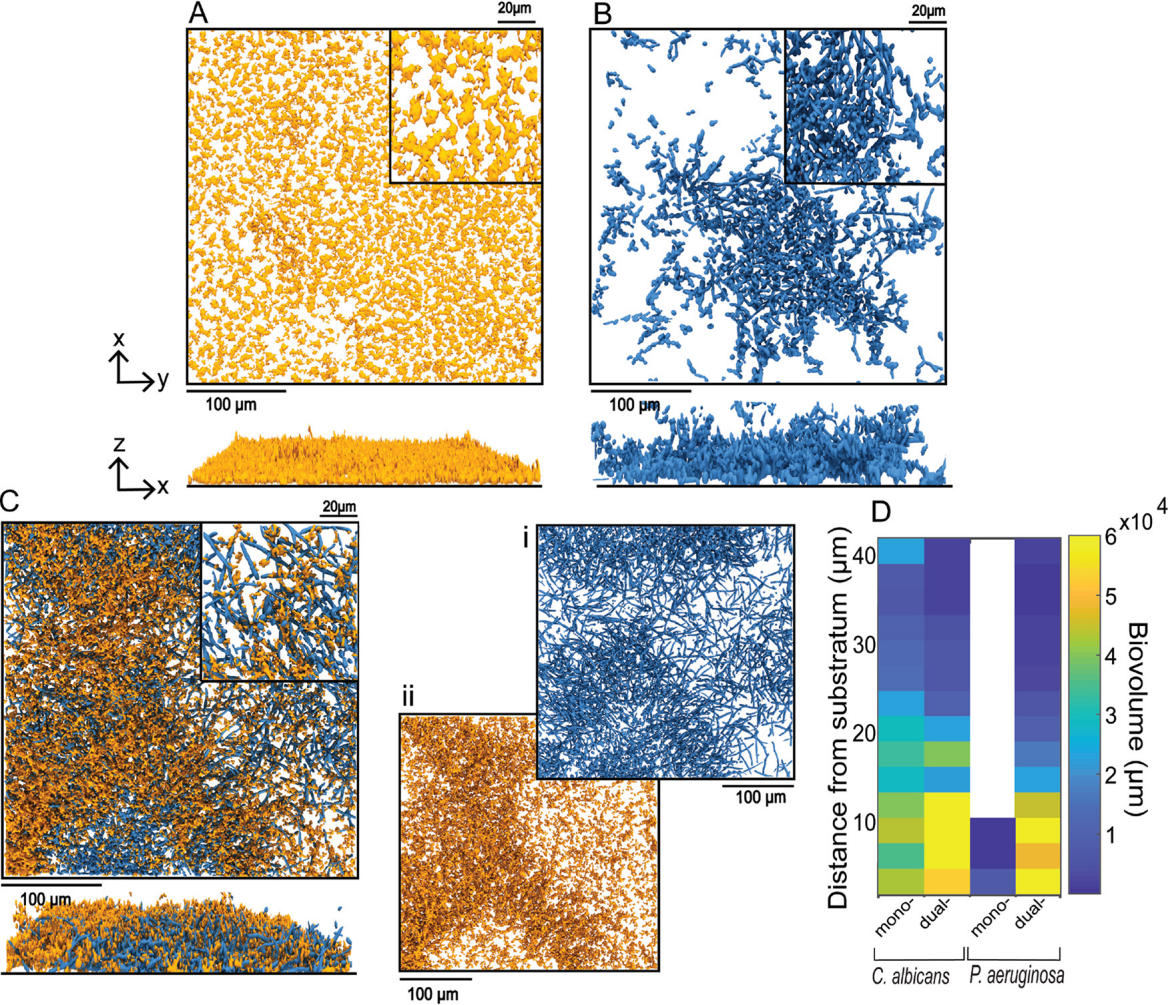
Representative images of mono- and dual-species biofilms of P. aeruginosa and C. albicans. Three-dimensional (3-D) renderings of 24-h-old monospecies biofilms of P. aeruginosa (A) and C. albicans (B). Bottom panels show side views of the same images as those above them. (C) P. aeruginosa*-*C. albicans dual-species biofilm at 24 h. Split channel of C. albicans biofilm (i) and P. aeruginosa biofilm (ii) from the P. aeruginosa*-*C. albicans dual-species biofilm. (D) Heat maps of C. albicans and P. aeruginosa biovolume as a function of height from the base substratum in mono- and dual-species biofilms from panels A to C.

To investigate mono- and dual-culture biofilm growth under flow of ASMi, we inoculated derivatives of P. aeruginosa strain PA14 and C. albicans strains CAI4 either alone or together in microfluidic devices (see Materials and Methods). Monococulture P. aeruginosa chambers contained small biofilms with compact microcolonies on the order of 10 μm in height (Fig. 1A). Monococulture biofilms of C. albicans contained scattered clusters of groups of elongated yeast, many pseudohyphae, and some true hyphae that spanned the height of the chamber (Fig. 1B). By visual inspection of confocal images, it was quickly clear that the architecture and total accumulation of both species were quite different in dual-inoculated conditions compared to the monococulture biofilms. In coculture, C. albicans had largely formed true hyphae (Fig. 1C). Quantification of C. albicans biovolume found a higher biovolume density near the base of the biofilm in coculture conditions (Fig. 1D). In coculture, P. aeruginosa biofilms localized to the hyphae of the highly filamentous C. albicans biofilms (Fig. 1C). The biovolume accumulation of P. aeruginosa in coculture appeared greater, particularly in the regions also colonized by C. albicans (0 to 12 μm from the glass substratum) (Fig. 1D).

**FIG 2:**
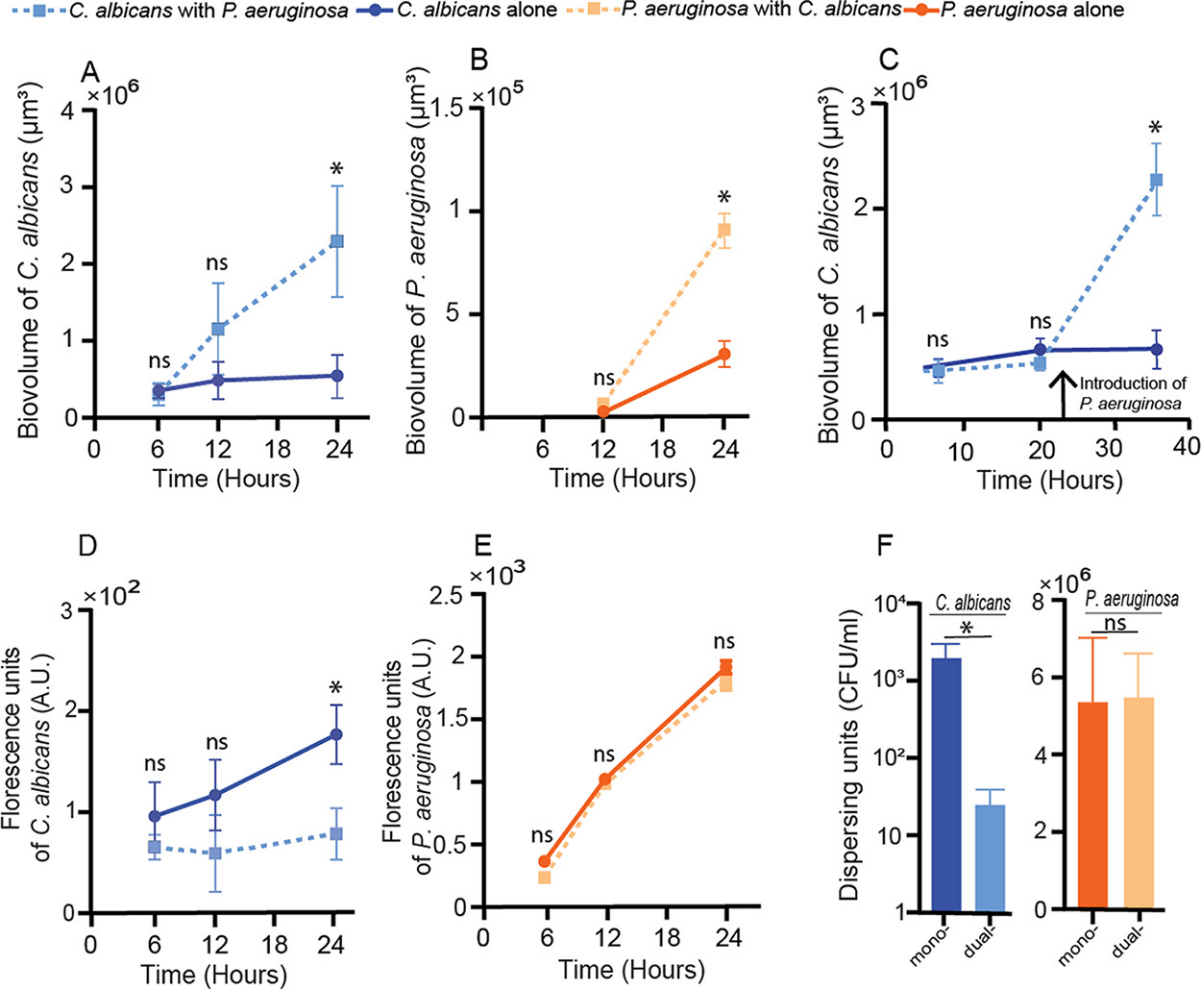
P. aeruginosa and C. albicans in mono- and dual-species culture. (A) Biovolume of C. albicans in mono- and dual-species biofilms (*n *= 24). (B) Biovolume of P. aeruginosa in mono- and dual-species biofilms (*n *= 24). (C) Biovolume of C. albicans biofilms initially grown in monococulture, with the addition of P. aeruginosa at the time point indicated by the vertical arrow. For the control, sterile medium was added in place of P. aeruginosa (*n *= 18). (D) Fluorescence counts of C. albicans in mono- and dual-species shaking liquid cultures (*n *= 10). (E) Fluorescence counts of P. aeruginosa (in arbitrary units [A.U.]) in mono- and dual-species shaking liquid cultures (*n *= 10). (F) Dispersing cells of P. aeruginosa and C. albicans in mono- and dual-species biofilms (*n *= 11). Error bars in panels A to E denote standard deviations; error bars in panel F denote standard errors. *, *P* < 0.05 by Wilcoxon signed-rank test with Bonferroni correction; ns, not significant.

Quantitative analysis of image stacks from replicate biofilms collected from independent experiments found that the total biovolume of both species increased substantially in coculture relative to monococulture (Fig. 2A and B). The increase in biofilm biovolume in coculture was significant by 24 h for C. albicans (Fig. 2A) and for P. aeruginosa (Fig. 2B). In order to determine whether the increase in biovolume required the presence of P. aeruginosa at the time of initial colonization, we added P. aeruginosa or a medium-only control to C. albicans 24-h-old biofilms. In these experiments, the P. aeruginosa cells were spiked into the chambers for 1 h, followed by a return to sterile ASMi medium. In control experiments, the same spiking procedure was performed but with sterile ASMi medium. While C. albicans biofilm accumulation followed its normal monococulture profile in the control condition, C. albicans biofilm development significantly increased over the subsequent 12 h after the introduction of P. aeruginosa (Fig. 2C). To determine whether any mechanical disturbance was sufficient to induce the increase in C. albicans biomass accumulation, we introduced 1-μm-diameter inert fluorescent beads to the chambers containing C. albicans, but we saw no change in biofilm architecture or biomass (Fig. S3).

10.1128/mSphere.00416-21.3 FIG S3 Inert fluorescent beads were introduced to C. albicans expressing *mKate2* biofilms to test for the effect of mechanical disturbance on C. albicans biofilm growth. (A) Biovolume of C. albicans with and without fluorescently labeled beads added to the influent medium (*n* = 6). (B) Representative image of C. albicans biofilms grown with fluorescently labeled beads shown in red at the 24-h time point (C) Representative image of C. albicans biofilm in the absence of fluorescent beads in influent medium at 24-h time point. All error bars indicated are standard errors. Download FIG S3, TIF file, 1.2 MB. Copyright © 2021 Kasetty et al. 2021 Kasetty et al. https://creativecommons.org/licenses/by/4.0/ This content is distributed under the terms of the Creative Commons Attribution 4.0 International license.

The enhancement of C. albicans biofilm volume by the presence of P. aeruginosa was not likely due to an overall improvement in growth when both species are present. In comparison experiments in which both organisms were cultivated planktonically in shaking liquid ASMi medium, the reverse pattern was seen for C. albicans: its population density was substantially lower in the presence of P. aeruginosa than in its absence (Fig. 2D), recapitulating previously established antagonistic C. albicans-P. aeruginosa interaction in liquid growth conditions (19, 28, 29). The population density of P. aeruginosa did not change in the presence of C. albicans in liquid culture (Fig. 2E). We infer from this outcome that the increase in accumulation of both species in microfluidic coculture is specific to the biofilm environment.

Because increased rate of biovolume increase can result from higher retention of cells in the chambers due to decreases in active dispersal or disruption by fluid flow, we quantified the cells in the effluent collected from the outlet of the microfluidic chambers (see Materials and Methods). Significantly fewer C. albicans cells were found in the effluent from dual-species biofilms (Fig. 2F). P. aeruginosa cell concentration in effluent stayed the same in absolute terms (Fig. 2F) but was lower upon normalization to the amount of biovolume in the biofilm chamber (Fig. S4).

10.1128/mSphere.00416-21.4 FIG S4 Passive dispersing P. aeruginosa CFU per unit biovolume. Dispersing P. aeruginosa obtained from plating cells from the outflow of the microfluidic chamber normalized to biovolume of cells present in the microfluidic chamber (*P* < 0.001; *n* = 6). Reported pairwise comparisons are the result of Wilcoxon signed-rank test. All error bars indicated are standard errors. Download FIG S4, TIF file, 0.1 MB. Copyright © 2021 Kasetty et al. 2021 Kasetty et al. https://creativecommons.org/licenses/by/4.0/ This content is distributed under the terms of the Creative Commons Attribution 4.0 International license.

### Exploration of P. aeruginosa genes potentially involved in augmenting C. albicans biofilms in coculture.

**FIG 3:**
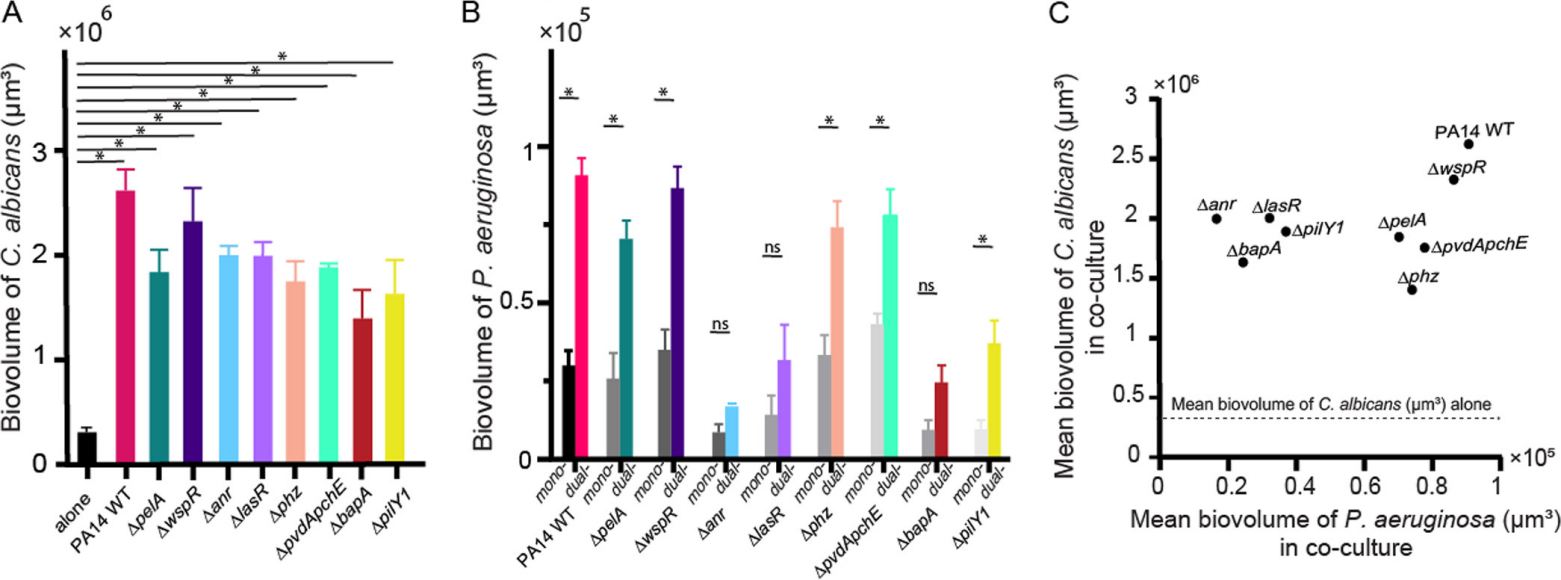
Deletion mutant assays and medium influent assays to explore the causes of mutual enhancement between P. aeruginosa and C. albicans in biofilms. (A) Biovolume of C. albicans grown in dual-species biofilms with P. aeruginosa deletion mutants at 24 h (see main text for mutant descriptions, *n* = 9 to 24). (B) Biovolumes of mono- and dual-species P. aeruginosa biofilms at 24 h (*n* = 9 to 24). All error bars indicated are standard errors. (C) The mean values of C. albicans biovolume are plotted against the corresponding mean value of wild-type (WT) P. aeruginosa PA14 and mutant biovolume from their respective dual-species biofilms. There is no significant correlation between the two (linear correlation analysis; *P* = 0.391; *r*^2^ = 0.107). *, *P* < 0.05 by Wilcoxon signed-rank test with Bonferroni correction.

We repeated the mono- and coculture experiments above with mutants of P. aeruginosa that have been implicated in altered biofilm morphology or interspecies interaction in prior work. Analyses included mutants defective in the Pel exopolysaccharide production (Δ*pelA* [30, 31] and Δ*wspR* [32]), metabolic regulators and products important for biofilm formation (Δ*anr* [33] and Δ*phz* [34]), extracellular adhesins (Δ*bapA* [35], Δ*pilY1* [36]), quorum sensing (Δ*lasR* [37]), and siderophore production (Δ*pvdApchE* [38]). C. albicans increased its accumulation by an order of magnitude or higher in biofilms with any of these mutants, maintaining the trend seen with wild-type P. aeruginosa PA14 (Fig. 3A and Fig. S5). In contrast, not all P. aeruginosa mutants were equal in their capacity for biofilm formation or for stimulation of biofilm biovolume in the presence of C. albicans (Fig. 3B). The Δ*pelA* and Δ*wspR* mutants were not defective in biofilm biovolume compared to the wild type, which is consistent with the low detection of Pel extracellular matrix carbohydrate (Fig. S6). Thus, the increased biovolume of P. aeruginosa was not due to increased P. aeruginosa matrix production. Previously characterized mutants defective in secreted phenazine toxins and pyochelin and pyoverdine siderophores also caused the stimulation of C. albicans biofilm accumulation. P. aeruginosa mutants with lower levels of monospecies biofilm (Δ*anr*, Δ*lasR*, Δ*bapA*, and Δ*pilY1*) were less stimulated by C. albicans at the 24-h time point. It is interesting to note that the amount of P. aeruginosa biofilm biomass present did not correlate with the degree of biomass increase in C. albicans (Fig. 3C); that is, any addition of P. aeruginosa, regardless of its native biofilm-producing capacity, was sufficient to produce a similar increase in accumulation of C. albicans.

10.1128/mSphere.00416-21.5 FIG S5 Slopes of C. albicans growth yields. Best fit lines were generated by fitting kinetic biofilm biovolume data by least square regression. The slopes of the best fit lines for growth yields of C. albicans grown in the presence of the P. aeruginosa mutants were statistically different from the slope determined for C. albicans grown alone. (*P* < 0.001 for slope of C. albicans with P. aeruginosa mutants compared to slope of 0 for C. albicans alone as determined by extra sum-of-squares F test.) Download FIG S5, TIF file, 0.3 MB. Copyright © 2021 Kasetty et al. 2021 Kasetty et al. https://creativecommons.org/licenses/by/4.0/ This content is distributed under the terms of the Creative Commons Attribution 4.0 International license.

10.1128/mSphere.00416-21.6 FIG S6 The matrix polysaccharide Pel was stained and quantified in P. aeruginosa strain PA14 biofilms using a fluorescent dye bound to Pel-specific lectin in M63* medium plus arginine, a biofilm assay medium [99]), and ASMi medium (*P* < 0.001; *n* = 6). Reported pairwise comparisons determined by Wilcoxon signed-rank test. All error bars indicated are standard errors. *5X M63 is as follows: 17.5 g dibasic potassium phosphate, 7.5 g monobasic potassium phosphate, 5.0 g ammonium sulfate into sterile 500 ml H_2_O. M63 is supplemented to obtain working concentrations of 0.4% l-arginine-monochloride and 1 mM MgSO_4_. Download FIG S6, TIF file, 0.1 MB. Copyright © 2021 Kasetty et al. 2021 Kasetty et al. https://creativecommons.org/licenses/by/4.0/ This content is distributed under the terms of the Creative Commons Attribution 4.0 International license.

### P. aeruginosa-C. albicans interaction is robust to CF isolate variation.

**FIG 4:**
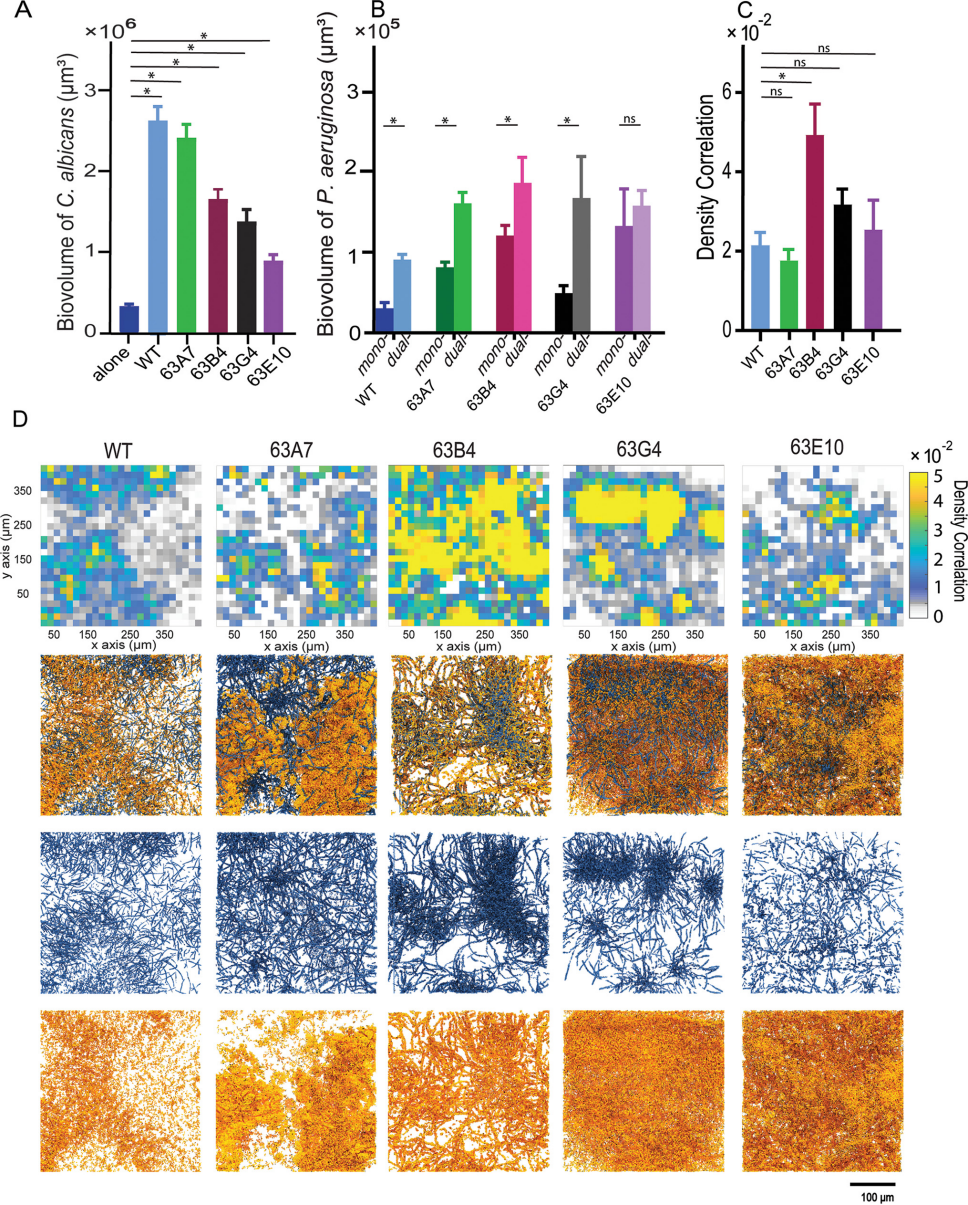
Biomass accumulation, density correlation analysis, and visualization of C. albicans in coculture with different CF clinical isolates of P. aeruginosa. (A) Biovolume of C. albicans grown as dual-species biofilms with P. aeruginosa clinical isolates along with wild-type (WT) PA14 for comparison at 24 h (*n* = 18). (B) Biovolumes of P. aeruginosa clinical isolates in monococulture and dual culture with C. albicans at 24 h (*n* = 18). (C) Global density correlation measurements of WT P. aeruginosa and clinical isolates and C. albicans biofilms (*n* = 6). *, *P* < 0.05. (D) Visualization of dual-species biofilms of P. aeruginosa and C. albicans. From top to bottom, spatially resolved density correlation, 3-D renderings of dual-species biofilms, C. albicans channel split, and P. aeruginosa channel split.

After documenting that wild-type PA14 could induce an increase in biofilm biomass accumulation of C. albicans, we were curious to see whether this effect was consistent across recent CF clinical isolates of P. aeruginosa as well. To explore this question, we obtained P. aeruginosa clinical isolates from a patient who was infected with both P. aeruginosa and C. albicans, and we grew them in mono- or coculture with C. albicans in our microfluidic model under flow of ASMi. We found that C. albicans biofilm increased significantly in coculture with all clinical isolates, consistent with the results reported above for wild-type PA14 (Fig. 4A). Likewise, for all but one isolate, the biofilm growth of P. aeruginosa was greater in coculture with C. albicans than it was in monococulture (Fig. 4B).

Though all clinical P. aeruginosa isolates prompted an increase in C. albicans biofilm accumulation, there was some variance in the degree to which this was the case (Fig. 4A). This variation made us wonder whether the spatial association between C. albicans and different clinical isolates of P. aeruginosa might differ as well. To assess this possibility, we grew the different clinical isolates together with C. albicans, acquired high-resolution images of coculture biofilms, and quantified the spatial cooccurrence of the two species via their density correlation (39). When averaged across all image replicates, the spatial correlations between C. albicans and clinical isolates of P. aeruginosa generally were not different from that between C. albicans and wild-type PA14 (Fig. 4C). After visualizing the density correlation measurement at high spatial resolution, on the other hand (Fig. 4D), it was clear that for some clinical P. aeruginosa isolates, the spatial association with C. albicans was homogenous, while for others it was patchy. Previous work has suggested that heterogeneity within a strain population—here, with respect to spatial cooccurrence with P. aeruginosa and C. albicans—can impact survival in variable environmental conditions (40–42). The significance of this result for the infection ecology of these two species is not yet clear, but it is notable that among isolates of P. aeruginosa from the same patient, the architecture of joint biofilms with C. albicans can differ substantially at the micrometer scale (Fig. 4D) even when they appear to be the same or similar when averaged on a larger spatial scale (Fig. 4C).

## DISCUSSION

Interest in multispecies biofilms including microbes from different domains of life has been intensifying in recent years, as it is increasingly appreciated that many microbial communities—both inside and outside host organisms—are polymicrobial (43). One of the most highly referenced examples of polymicrobial infections are those within the lungs of patients with CF, and two of the common members of these communities are the opportunistic pathogens P. aeruginosa and C. albicans (12). Here, we sought to compare the kinetics of biovolume accumulation in mono- and dual-species biofilms of these two organisms using a new model of biofilm growth under flow of optically clear artificial sputum medium. We demonstrated a marked increase of biofilm biomass accumulation as well as a decrease in cells in biofilm effluent in dual-species culture relative to monococulture. These results were robust to a variety of mutant and clinical strain backgrounds of P. aeruginosa, and they contrast with the findings of some previous studies of these two organisms in static liquid or agar colony culture (44, 45). We identify an important element driving the increase in biomass accumulation as fluid flow in the dual-species biofilm milieu, which is a key novelty of this experimental approach for the study of P. aeruginosa-C. albicans interactions.

Extensive prior work has shown that P. aeruginosa and C. albicans interact with each other through a complex web of secreted factors, including phenazines, siderophores, ethanol, and quorum-sensing autoinducers, which altogether alter environmental iron availability, pH, and oxygen tension. Under static culture conditions (i.e., liquid batch culture or agar colonies), the net result of these interactions is usually antagonism of P. aeruginosa against C. albicans. It is important to note as well that secreted factors from each species have different and sometimes opposite effects on each other’s propensity to produce biofilms or to remain in a dispersive, planktonic state (28, 46). As noted above, when flow—known to impact microbial physiology and surface interaction—is introduced into the two-species system, we see increased filamentation of C. albicans and increased biofilm biomass accumulation by both species, accompanied by a decrease in cells exiting the chamber.

While at first glance this may give the impression of mutual benefit, it is also possible that the two species are simply competing for access to space and resources by upregulating adhesion factors (47–49). But why is P. aeruginosa no longer able to directly antagonize and kill C. albicans, as has been shown previously in static culture? We speculate that introduction of flow fundamentally changes the secreted solute environment created by the two organisms, perhaps with some secreted factors more strongly retained in the biofilm matrix than others, and that this change in solute environment relative to static culture shifts the ecological pattern of biomass accumulation to one in which both species are augmented. It is also possible that over time the dual-species biofilms become densely packed enough to block flow within some regions, allowing secreted products and variation in iron/oxygen availability to accumulate in a patchy manner that contributes to induction of biofilm production by both species. The precise spatial patterns of exoproduct accumulation in relation to cells and the highly complex matrix that *Candida* secretes is an important area for future work (50–52).

Our deletion mutant analysis included all the major classes of behavior in P. aeruginosa currently known to mediate solute-based interactions with C. albicans, but in all cases, the presence of P. aeruginosa caused qualitatively the same increase in C. albicans biofilm. This suggests that there may be other factors in addition to flow-mediated changes in solute environment contributing to our results. For example, the introduction of shear stress under flow is an entirely new environmental stimulus relative to static culture, and one which is known via extensive work to be crucial to microbial ecology and evolution (53–58). The flow regime can dramatically alter the morphology and resilience of bacterial biofilms down to their cellular resolution architecture (59, 60), with important implications for pathogenesis in the case of infections (61). Adaptation to the challenges of flow at submillimeter spatial scales has influenced the evolution of bacterial surface motility (2), optimal growth rate in porous media (62), surface colonization mechanisms (63–65), extracellular matrix secretion (66, 67), bacterial cell shape (64, 68–70), planktonic aggregate formation (71), and biofilm community assembly and function (62, 72–75), among many other examples.

The range of spatial structures of P. aeruginosa clinical isolates that we observed in dual-species biofilms with C. albicans suggests the possibility of between-strain variance in spatial occupation strategy within the CF lung. Since the clinical isolates come from a single CF patient, this variation in biofilm morphology could be the outcome of selection in different spatial locations in the lung, which may have variable C. albicans abundance or exposure to antibiotics, toxins, mutagens, nutrient availability, or host immune attack (40, 41). Although the increase in biovolume of both species in dual P. aeruginosa-C. albicans biofilms varied to an extent, increase of C. albicans accumulation was consistent across P. aeruginosa isolates. This result prompts us to speculate that the chance encounter of C. albicans with P. aeruginosa in the CF environment could ultimately lead to changes in disease progression by altering the tendency of the fungus to locally accumulate.

In light of our results, it is important to note that the flow regime has documented effects on biofilm formation for both P. aeruginosa and C. albicans. The surface residence time of P. aeruginosa, for example, increases linearly as shear stress increases (76), and flow promotes upstream surface motility in addition to the formation of biofilm aggregates (77). P. aeruginosa has also recently been shown to be highly responsive to mechanical stress induced by flow, with downstream effects on biofilm formation that have yet to be fully clarified (36, 78). There has been less investigation of the effects of shear flow on C. albicans biofilms: existing work does not agree completely on whether shear stress increases total biomass of C. albicans biofilms but does agree that biofilms formed under shear are more highly compacted and physically robust relative to those grown in static conditions (79). Importantly, given that dual-species culture produced substantial biomass accumulation for both species relative to monococulture under the same flow conditions, flow-induced shear cannot on its own explain our results. Rather we infer that a combination of physical forces resulting from flow in addition to biological interaction between the two species must be responsible for the results obtained here. Dissecting the precise molecular mechanisms of these interspecies interactions is an important area for future study that may bear directly on the outcome of multispecies biofilm growth in the context of infection.

Beyond their prevalence in lung infections among patients with CF, P. aeruginosa and C. albicans individually are among the most common agents of nosocomial infection currently known (16). They are both frequently isolated from device-related infections, including implanted medical devices, prosthetic implants in wounds and joint replacements, and urinary catheters (16). Both species participate in multispecies infections, for example, with Staphylococcus spp. (80–82), with Streptococcus spp. (83, 84), and with each other (85). Reports of dual isolation of P. aeruginosa and C. albicans are increasingly reported in the clinical literature in sites such as ventilator tubing (86), and our results of biofilm dual-species culture in microfluidic devices suggest that dual Pseudomonas-*Candida* biofilms may be especially problematic in this setting because they tend to accumulate more biofilm biomass together than alone. Such rapidly accumulating biofilms can potentially clog catheter flow environments and seed systemic infections as cells disperse from the device-attached biofilm into the bloodstream.

Though recent studies have made tremendous strides in imaging microbiomes within *in situ* samples that have been fixed (87–90), dissecting live microbial community structure in space and time within native environments remains a challenging task and one of the important frontiers of modern microbiology. Here, we use an *in vitro* model with medium tuned to the CF sputum environment to assess live biofilm population dynamics for both members and find that this step toward environmental realism has a strong impact on the ecology of dual-species biofilms of P. aeruginosa and C. albicans. Many native factors are still missing, however: the mucosal environment is quite different in the native lung, for example, and recent work has suggested that mucus has a strong impact on P. aeruginosa physiology, including reducing its propensity toward virulence and biofilm formation (91, 92). Though not an exact match to the *in situ* infection environment, our system nevertheless suggests that modest changes to the environmental context in which multispecies interactions are studied can have a large impact on the observed outcome, namely, in this case, a shift toward far higher accumulation of biofilm on the part of P. aeruginosa and C. albicans when they are together versus when they are alone. On the basis of this observation, we speculate that pushing toward realism and high-resolution image analysis of biofilm communities will yield important and unexpected insights for many other microbial systems of interest.

## MATERIALS AND METHODS

### Strains and media.

Table 1 includes a full list of strains and plasmids used in this study. Strains of P. aeruginosa are either derivatives of strain PA14 or clinical isolates. C. albicans strains are derivatives of strain CAI4. All strains were grown on LB (10 g tryptone, 5 g NaCl, 5 g yeast extract [all amounts per liter]) and artificial sputum medium for imaging (ASMi) (P. aeruginosa) or YPD (10 g yeast extract, 20 g peptone, and 20 g dextrose [all amounts per liter]) and ASMi (C. albicans). The medium recipes and concentrations of reagents used for ASMi are listed below at the end of Materials and Methods. All chemicals and reagents were purchased from Millipore Sigma unless otherwise stated.

**TABLE 1:** Strains and plasmids used in this study

Species and strain	Relevant marker(s) or genotype(s)	Reference or source
E. coli		
S17-1	λ*pir*	Lorenzo and Timmis (100)
P. aeruginosa PA14		
CNP17	Wild type (WT)	Hogan lab
CNP26	WT with mKO-κ	This study
CNP12	Δ*pelA*	Friedman and Kolter (30)
CNP27	Δ*pelA* with mKO-κ	This study
CNP18	*ΔwspR*	Chen et al. (21)
CNP28	Δ*wspR* with mKO-κ	This study
CNP70	Δ*bapA*	Hogan lab
CNP77	Δ*bapA* with mKO-κ	This study
CNP65	Δ*pilY1*	Hogan lab
CNP67	Δ*pilY1* with mKO-κ	This study
CNP21	Δ*anr*	Hogan lab
CNP50	Δ*anr* with mKO-κ	This study
CNP69	Δ*pchEpvdA*	Hogan lab
CNP76	Δ*pchEpvdA* with mKO-κ	This study
CNP22	Δ*lasR*	Hogan lab
CNP56	Δ*lasR* with mKO-κ	This study
CNP41	Δ*phz*	Hogan lab
CNP54	Δ*phz* with mKO-κ	This study
CNP43	63LB4 clinical CF isolate	Hogan lab
CNP44	63LG4 clinical CF isolate	Hogan lab
CNP45	63RA7 clinical CF isolate	Hogan lab
CNP46	63RE10 clinical CF isolate	Hogan lab
CNP59	63LB4 clinical CF isolate with mKO-κ	This study
CNP57	63LG4 clinical CF isolate with mKO-κ	This study
CNP60	63RA7 clinical CF isolate with mKO-κ	This study
CNP58	63RE10 clinical CF isolate with mKO-κ	This study
C. albicans CAI4		
CNC1	WT with pACT-GFP	Hogan lab
CNC11	WT with pACT mKATE2	This study

### Plasmid and strain construction.

All restriction enzymes and ligase were purchased from New England Biolabs, and PCR reagents were purchased from Bio-Rad. The P. aeruginosa tandem codon-optimized version of *mKO*-κ was custom synthesized by Invitrogen. The construct contains two copies of *mKO*-κ in tandem, each with its own ribosome binding site, and with different codon composition to prevent excision by recombination. Fluorescent P. aeruginosa derivatives were constructed by amplification of the flanking regions upstream and downstream of the Tn*7 att* site and fusion of the custom fluorescent protein construct to a synthetic *tac* promoter for high expression from a single chromosomal locus. This fused construct was cloned into the pMQ30 plasmid used for allelic exchange in P. aeruginosa (93). This plasmid was then introduced into Escherichia coli S17-λ*pir* by electroporation and conjugated into P. aeruginosa, and recombinants were obtained using selection on gentamicin and sucrose counterselection for loss of the integrated plasmid backbone. For C. albicans, a single codon-optimized version of *mKate2* was custom synthesized by Invitrogen. The RP10 integrative plasmid, pACT-GFP (94), has been shown to have constant expression levels through C. albicans growth cycle. We replaced the green fluorescent protein (GFP) in pACT-GFP (94) with *mKate2.* For transformation into C. albicans, the *mKate2-*containing plasmid was linearized by BglII restriction digestion and concentrated using the Zymo Research DNA Clean & Concentrator-5 kit (catalog no. 11-303), and 1 μg was electroporated into electrocompetent C. albicans CAI4 prepared as previously described (95). Prototrophic recombinants were selected for on uracil drop-out medium.

### Liquid growth curve and fluorescence measurements.

P. aeruginosa strains were grown at 37°C shaking in LB overnight prior to growth curve experiments. The following morning, cultures were back-diluted to an optical density at 600 nm (OD_600_) of 0.01 in ASMi in 10-ml glass tubes with 2 ml medium (for fluorescence growth curves) or 50-ml Falcon tubes with 30 ml of medium (optical density growth curves), rotating at 250 rpm on an incubated orbital shaker at 37°C. C. albicans strains were grown at 30°C shaking in YPD overnight prior to growth curve experiments. They were cultivated overnight at 30°C to maintain cells in yeast form prior to the start of growth curve or biofilm experiments (see below). The following morning, cultures were back-diluted to an OD_600_ of 0.01 in ASMi in 10-ml glass tubes with 2 ml medium (for fluorescence growth curves) or 50-ml Falcon tubes with 30 ml of medium (optical density growth curves), rotating at 250 rpm on an incubated orbital shaker at 37°C. Fluorescence measurements were made using a Synergy Neo2 every 6 h. A 543-nm excitation source was used to excite *mKO-κ*, and a 594-nm excitation source was used to excite *mKate2*. Optical density measurements were made every hour using a benchtop spectrophotometer (CWA Biowave CO8000 cell density meter).

### Microfluidic device assembly.

The microfluidic devices were made by bonding polydimethylsiloxane (PDMS) chamber molds to size #1.5 cover glass slips (60 mm × 36 mm [length *L* × width *W*], Thermo-Fisher, Waltham, MA) using standard soft lithography techniques (96). Each PDMS mold contained four chambers, each of which measured 3,000 μm × 500 μm × 75 μm (*L* × *W* × depth *D*). To establish flow in these chambers, medium was loaded into 1-ml BD plastic syringes with 25-gauge needles. These syringes were joined to #30 Cole-Parmer polytetrafluoroethylene (PTFE) tubing (inner diameter, 0.3 mm), which was connected to prebored holes in the microfluidic device. Tubing was also placed on the opposite end of the chamber to direct the effluent to a waste container. Syringes were mounted to syringe pumps (Pico Plus Elite, Harvard Apparatus), and flow was maintained at 0.1 μl per min for all experiments.

### Biofilm growth, matrix staining, and CFU counts.

Overnight cultures of P. aeruginosa were grown at 37°C with shaking in LB, and overnight cultures of C. albicans were grown at 30°C with shaking in YPD prior to the start of biofilm experiments. Cultures of both strains were normalized to an OD_600_ of 0.05 in ASMi medium. If dual-species biofilms were to be started, equal volumes of OD-equalized strains were mixed, inoculated into a microfluidic chamber (completely filling its inner volume), and then allowed to rest for 1 h at 37°C to permit cells to attach to the glass surface. The devices were then run at 0.1 μl per min at 37°C and imaged by confocal microscopy (see below) at time intervals that varied per experiment as noted in each figure. All experiments were repeated with at least five biological replicates with three or more technical replicates on different days. Total replicates for each experiment are noted in the figure legends for each data set in the text and supplemental material.

Wisteria floribunda lectin stain (Vector Labs) conjugated to fluorescein dye was used to visualize Pel polysaccharide produced by P. aeruginosa (31). The lectin was added to the medium in syringes for these experiments such that biofilms would be exposed to the lectin-dye conjugate for the entire period of biofilm growth (20 μl stock lectin solution per ml of medium, per the manufacturer’s protocol recommendation from a stock solution of 2-mg/ml dye conjugate). Biofilms were inoculated as noted above for these experiments and grown for 24 h prior to imaging.

To compare growth rates of P. aeruginosa and C. albicans in turbid synthetic cystic fibrosis medium (SCFM) (25) and optically clear ASMi, both species were grown overnight, P. aeruginosa in LB at 37°C and C. albicans in YPD at 30°C in 10-ml glass tubes with 2 ml of medium. The following morning, the cultures were back-diluted to an OD_600_ of 0.01 in either SCFM or ASMi in 50-ml Falcon tubes with 30 ml of medium, rotating at 250 rpm in an orbital shaker at 37°C. One milliliter of culture was taken from the Falcon tube at different time points, and serial dilution was performed and plated on LB agar for P. aeruginosa and YPD agar for C. albicans. The number of CFU from each plate was recorded and used to calculate growth rates measured by CFU per milliliter per time.

To measure passive dispersal from biofilms as a result of exposure to fluid shear, biofilms of both species were grown as noted above in ASMi medium for 24 h, after which the outlet tubing of the microfluidic device was changed to ensure we were measuring dispersal only from the biofilms within the chambers themselves. The flow rate was increased to 500 μl per min, and outflow was collected. Serial dilutions were performed and plated on LB agar for P. aeruginosa and YPD containing 50 μg/ml chloramphenicol for C. albicans. The number of CFU from each plate was recorded and used to calculate the CFU/milliliter culture density emerging from the chambers. This experiment was repeated for 11 biological replicates with independent overnight cultures.

### Microscopy and image analysis.

Biofilms inside microfluidic chambers were imaged using a Zeiss LSM 880 confocal microscope with a 40×/1.2 numerical aperture (NA) or 10×/0.4 NA water objective. A 543-nm laser line was used to excite mKO-κ, and a 594-nm laser line was used to excite mKate2. A 458-nm laser line was used to excite *Wisteria floribunda* lectin stain in the case of Pel quantification experiments. All quantitative analysis of microscopy data was performed using BiofilmQ (39). Three-dimensional (3-D) renderings of biofilms in Fig. 1 and 4 were made using Paraview.

### Statistics.

All statistical analyses were performed in GraphPad Prism. All reported pairwise comparisons were performed using Wilcoxon signed-rank tests, and multiple comparisons were performed by Wilcoxon signed-rank tests with Bonferroni correction. All error bars indicated standard errors unless otherwise noted.

### Artificial sputum media for imaging (ASMi).

The stocks for the base were Na_2_HPO_4_ (0.2 M, 0.69 g/25 ml), NaH_2_PO_4_ (0.2 M, 0.71 g/25 ml), KNO_3_ (1 M, 2.53 g/25 ml), K_2_SO_4_ (0.25 M, 1.09 g/25 ml). Additional stocks were glucose (20% [wt/vol]) autoclave, l-lactic acid (1 M) (adjust pH to 7 with NaOH), CaCl_2_·2H_2_O (1 M, 3.68 g/25 ml), MgCl_2_·6H_2_O (1 M, 5.08 g/25 ml), FeSO_4_·7H_2_O (1 mg/1 ml) syringe, *N*-acetylglucosamine (0.25 M, 1.383 g/25 ml), tryptophan (0.1 M, 1.021 g/50 ml). Reagents were DNA (herring sperm DNA), fucose, GalNAc, galactose, choline chloride, sodium octanoate, yeast synthetic dropout excluding Trp, NaCl, morpholinepropanesulfonic acid (MOPS), KCl, NH_4_Cl, and NaOH. Preparation of ASMi (500 ml) (2× in 250 ml) was as follows: 1) add 400 ml distilled H_2_O (diH_2_O) and stir bar to a clean beaker; 2) while stirring add 3.250 ml Na_2_HPO_4_ stock, 3.126 ml NaH_2_PO_4_ stock, 174 μl KNO_3_ stock, 542 μl K_2_SO_4_ stock, 2 g yeast synthetic dropout – Trp, 1.516 g NaCl, 1.046 g morpholinepropanesulfonic acid (MOPS), 558 mg KCl, 62 mg NH_4_Cl, 4.65 ml l-lactic acid stock, 1.365 ml glucose stock, 875 μl CaCl_2_·2H_2_O stock, 600 μl *N*-acetylglucosamine, 500 μl FeSO_4_·7H_2_O, 330 μl tryptophan stock, 303 μl MgCl_2_·6H_2_O, 300 mg DNA, 0.007 g choline chloride, 0.022 g sodium octanoate (replacement 1,2-dipalmitoyl-*sn*-glycero-3-phosphocholine [DPPC]), 400 mg fucose, 125 mg GalNAc, 90 mg galactose, (replacement for mucin; these are mucin sugars); 3) adjust pH to 6.8 with HCl or NaOH and add distilled H_2_O to 500 ml; 4) filter sterilize.

### Considerations and references.

Considerations follow. (i) It lacks sphingolipids and surfactant proteins, which are moderately abundant. (ii) Mucin sugars are used instead of mucin (97). (iii) Reports of some concentrations vary from source to source. References follow: DPPC (98) (octanoate and choline are used instead at the same concentrations; 2:1 octanoate-choline, since DPPC has two lipid chains per choline. DPPC molarity for choline and 2× that for octanoate), DNA (98), and mucin (98).
